# Supplementary material for: GIGEM (Group Isolation Gauge Effect Metrics), A Software Suite for Analyzing Social Isolation-Induced Sleep Loss and Multi-Batch Experiments in Drosophila
Source: bioRxiv. 2025 Dec 22:2025.12.18.695326. Preprint. [Version 1] doi: 10.64898/2025.12.18.695326 (PMC12776143; doi:10.64898/2025.12.18.695326)
Supplement: Supplement 1 [file NIHPP2025.12.18.695326v1-supplement-1.pdf]

bioRxiv preprint doi: https://doi.org/10.64898/2025.12.18.695326; this version posted December 22, 2025. The copyright holder for this preprint (which was not certified by peer review) is the author/funder, who has granted bioRxiv a license to display the preprint in perpetuity. It is made available under aCC-BY 4.0 International license.

Supplementary Materials

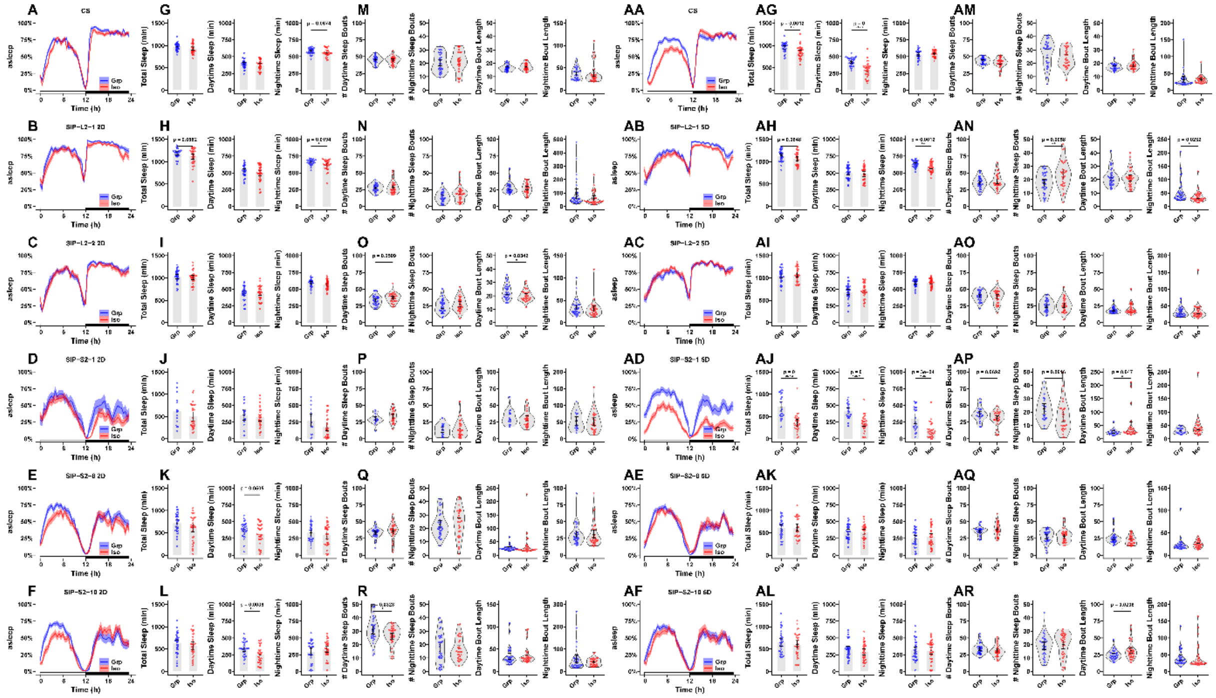

Supplementary Figure 1. Sleep profiles and sleep parameters for a single experimental batch. **(A–F; AA–AF)** Population sleep profiles show the percentage of animals asleep in 30-minute intervals, averaged across 2 LD cycles after isolation treatment of 2 days (A-F) or 5 days **(AA–AF)**. Solid lines represent the mean; shaded areas indicate ±SEM. Each data point represents an individual animal's average for the indicated sleep parameter, including: total sleep (ZT0–24) daytime sleep (ZT0–12), and nighttime sleep (ZT12–24) **(G–L; AG–AL)**, as well as the number of daytime sleep bouts, the number of nighttime sleep bouts, average daytime bout length, and average nighttime bout length **(M–R; AM–AR)**. Social isolation-induced sleep changes after 2 days of social isolation are shown on the left. Social isolation-induced sleep changes after 5 days of social isolation are shown on the right. Statistical significance was assessed by two-sided unpaired *t*-tests (**P<0.05, **P<0.01, ***P<0.001, ****P<0.0001*; *n* = 15–32 flies.

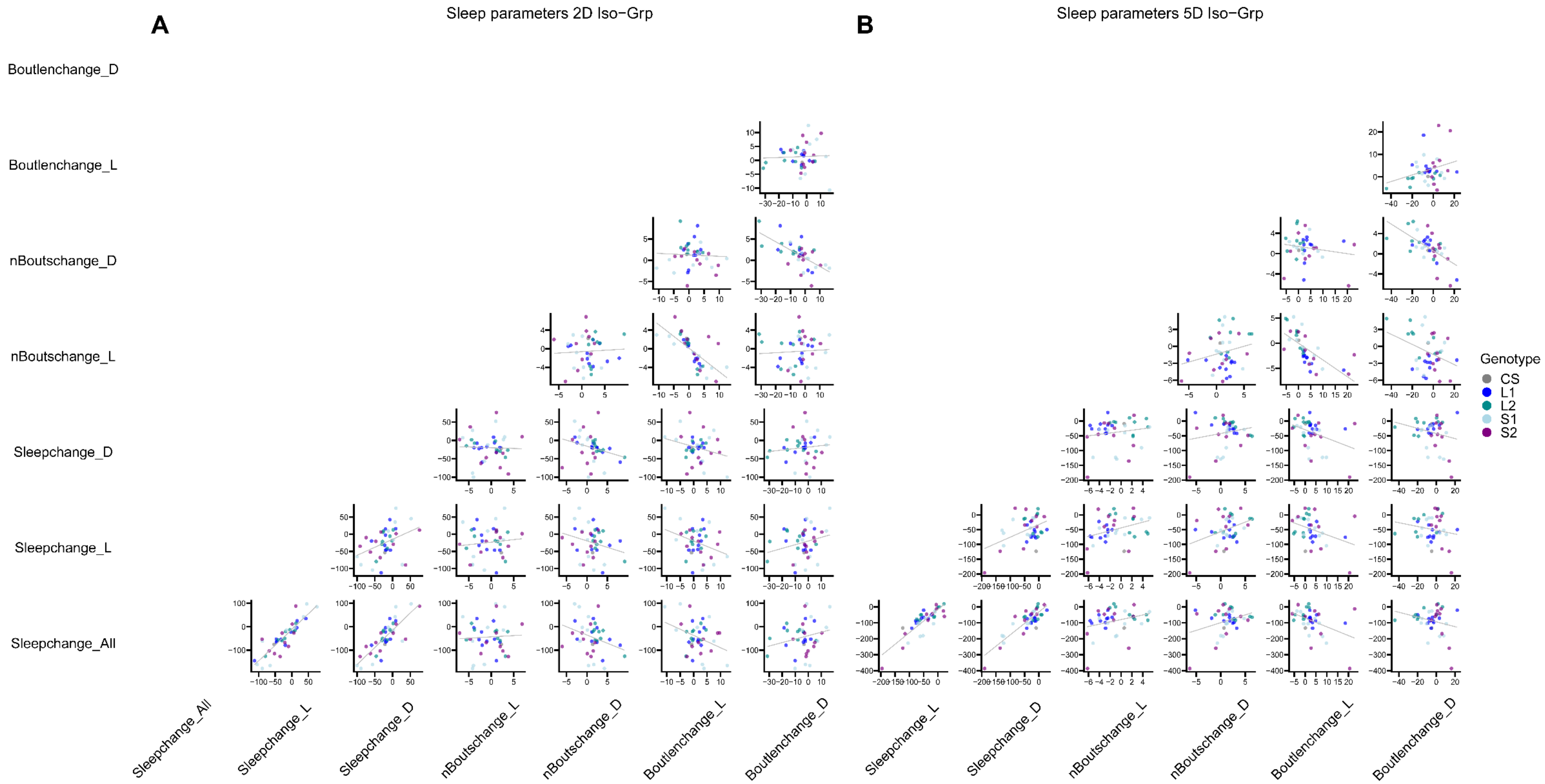

Supplementary Figure 2. Scatterplot visualization of correlation matrix data (Figure 3). The data was filtered for outliers, fit to a linear model, and adjusted for batch effect to generate predicted sleep values for each strain. Panels **A** and **B** display a matrix of scatterplots comparing the difference between isolated and group-housed averages (i − g) for each parameter by strain. Each point represents the strain-level mean for 38 Sleep Inbred Panel (SIP) lines and *Canton S* (CS). Linear regression lines are overlaid to indicate the direction and center-line of the relationships between traits. Colors represent genetic background. Data is divided by social isolation duration for 2 days (A) or 5 days (B). These plots visualize the same data shown in the correlation matrices of Figure 3, with scatterplot positions corresponding directly to the correlation matrix's boxed r-value position. n = 39 strain

bioRxiv preprint doi: https://doi.org/10.64898/2025.12.18.695326; this version posted December 22, 2025. The copyright holder for this preprint (which was not certified by peer review) is the author/funder, who has granted bioRxiv a license to display the preprint in perpetuity. It is made available under aCC-BY 4.0 International license.

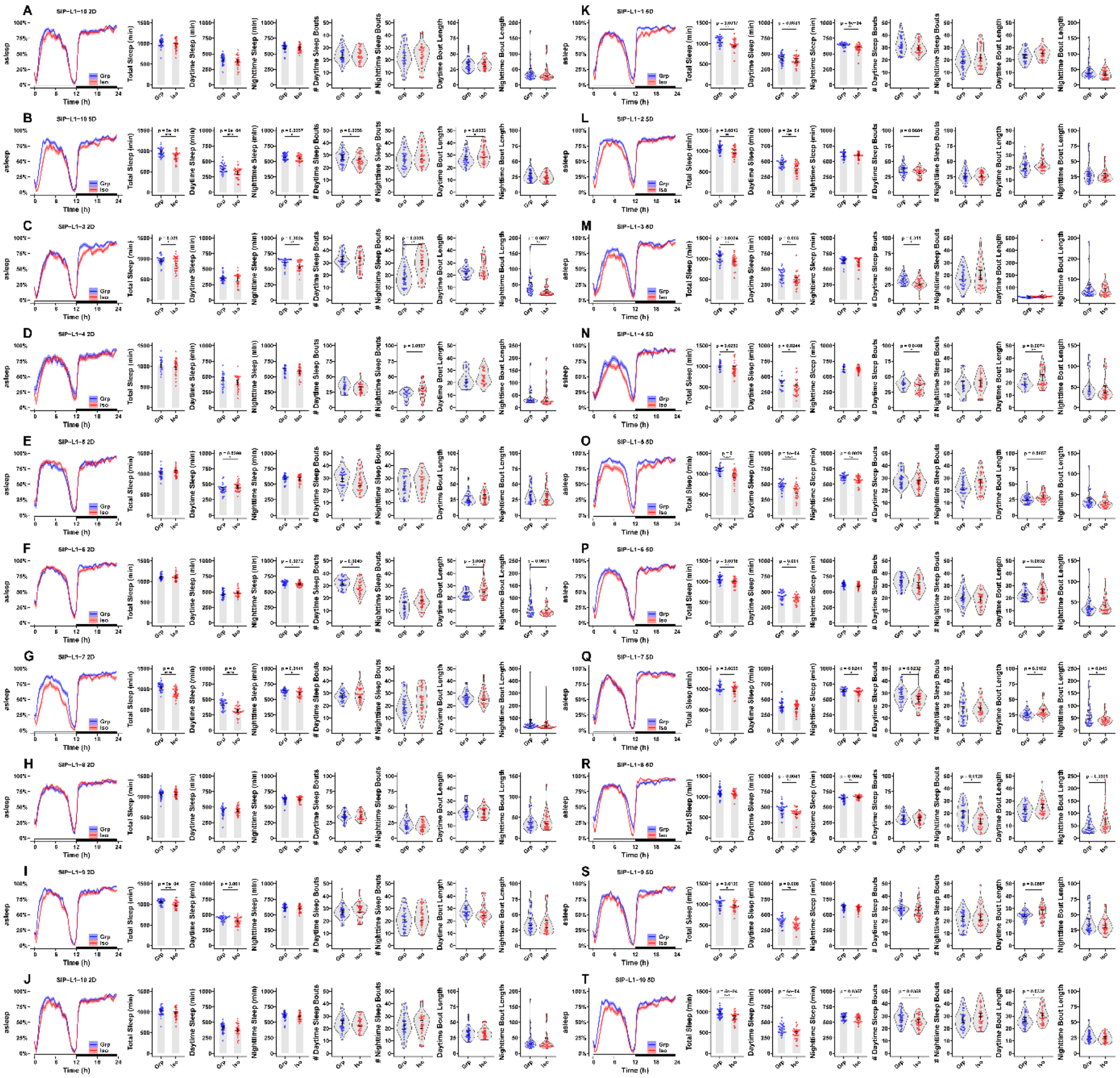

Supplementary Figure 3. Sleep profiles and parameters for SIP-L1 Strains. **(A–T)** SIP-L1 strains 1–10's sleep profiles, sleep parameters, and bout distributions after 2 days **(A–J)** or 5 days **(K–T)** of treatment. Statistical significance was assessed by two-sided unpaired *t*-tests (**P<0.05, **P<0.01, ***P<0.001, ****P<0.0001*; *n* = 17–32).

bioRxiv preprint doi: https://doi.org/10.64898/2025.12.18.695326; this version posted December 22, 2025. The copyright holder for this preprint (which was not certified by peer review) is the author/funder, who has granted bioRxiv a license to display the preprint in perpetuity. It is made available under aCC-BY 4.0 International license.

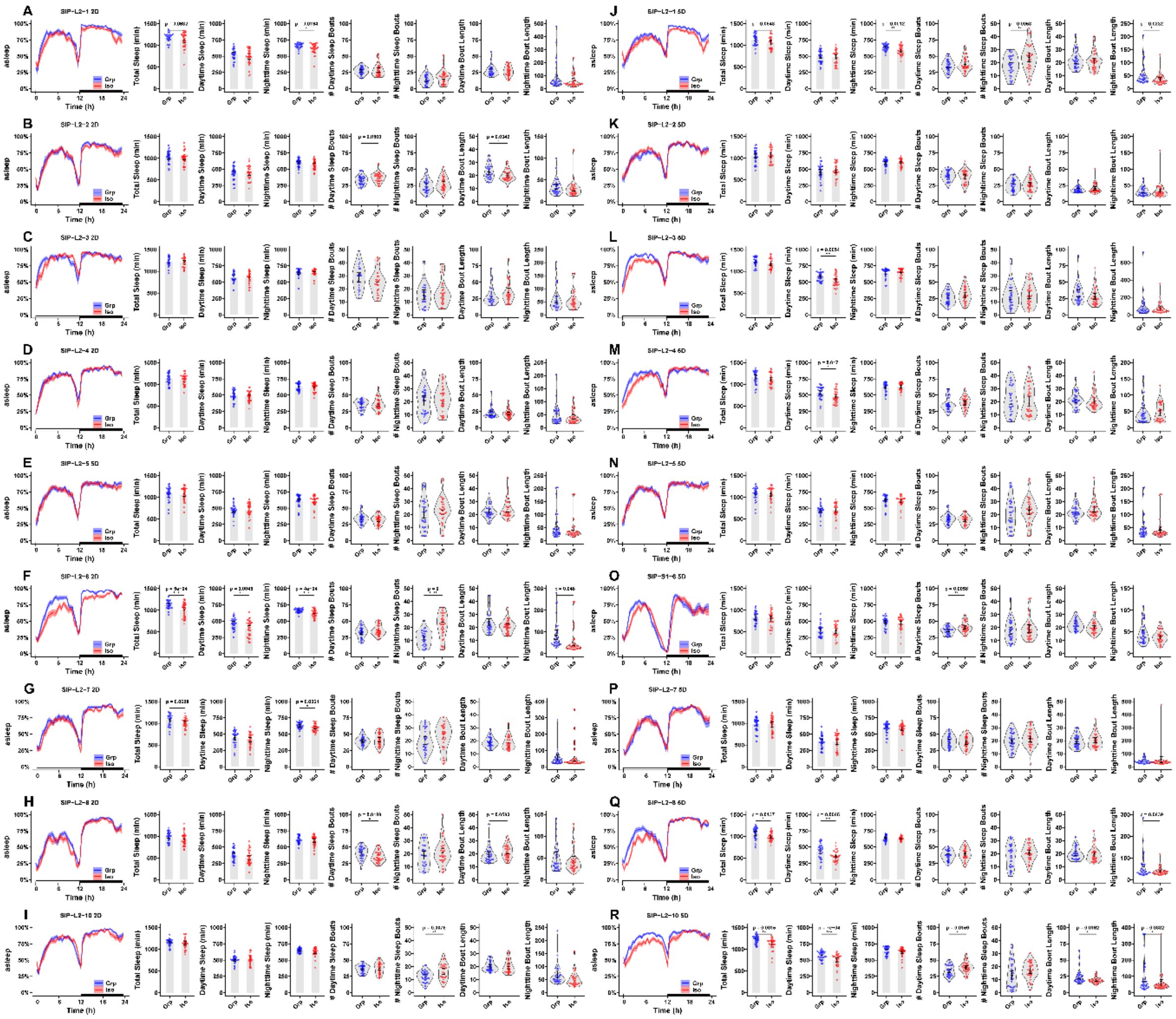

Supplementary Figure 4. Sleep profiles and parameters for SIP-L2 Strains. **(A–R)** SIP-L2 strains 1–8, and 10's sleep profiles, sleep parameters, and bout distributions after 2 days **(A–I)** or 5 days **(J–R)** of treatment as described. Statistical significance was assessed by two-sided unpaired *t*-tests (**P<0.05, **P<0.01, ***P<0.001, ****P<0.0001*; n = 25–32).

bioRxiv preprint doi: https://doi.org/10.64898/2025.12.18.695326; this version posted December 22, 2025. The copyright holder for this preprint (which was not certified by peer review) is the author/funder, who has granted bioRxiv a license to display the preprint in perpetuity. It is made available under aCC-BY 4.0 International license.

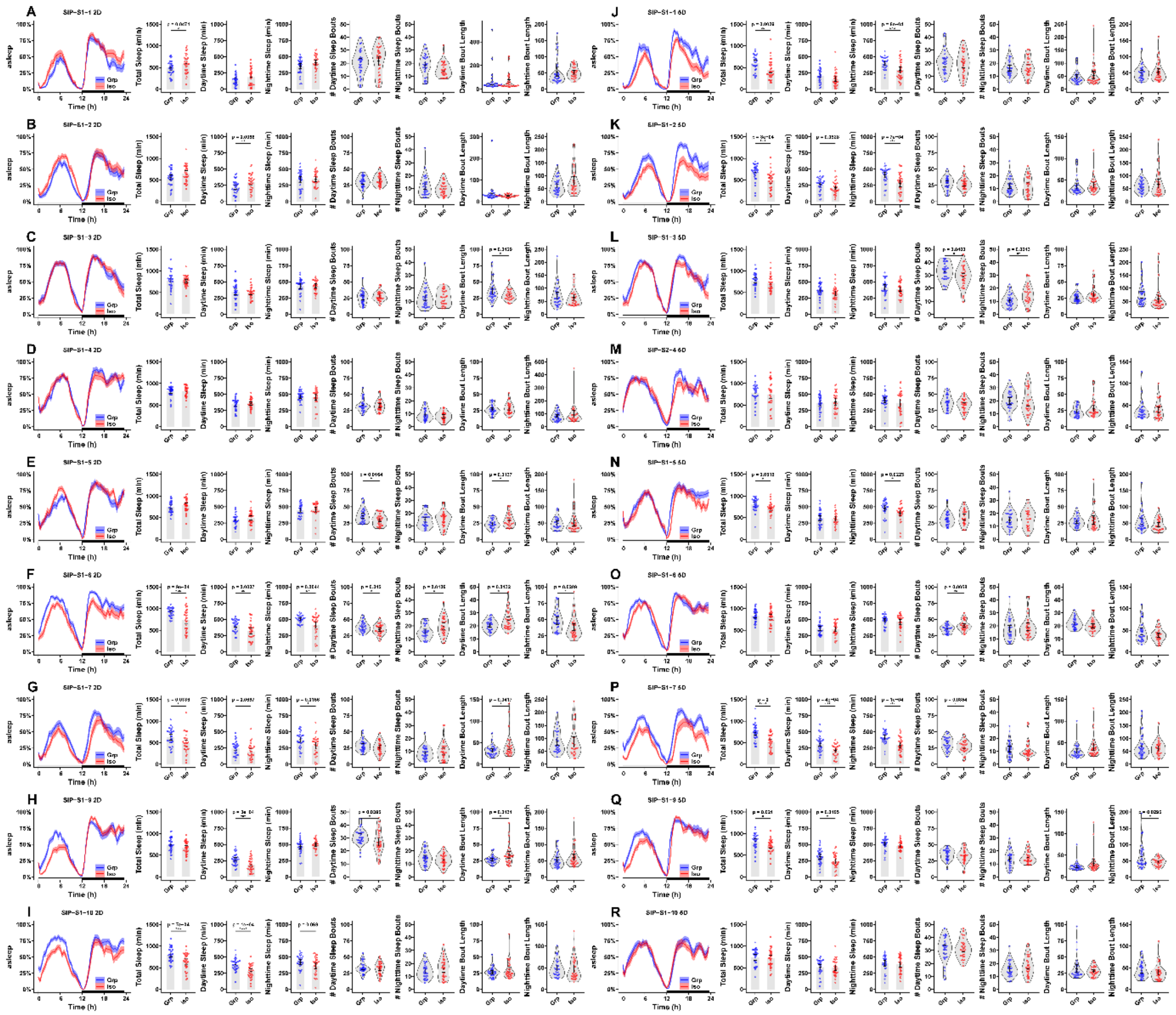

Supplementary Figure 5. Sleep profiles and parameters for SIP-S1 Strains. **(A–R)** SIP-S1 strains 1–7, and 9–10's sleep profiles, sleep parameters, and bout distributions after 2 days **(A–I)** or 5 days **(J–R)** of treatment. Statistical significance was assessed by two-sided unpaired *t*-tests (*$*P<0.05$, $**P<0.01$, $***P<0.001$, $****P<0.0001$*; n = 26–32).

bioRxiv preprint doi: https://doi.org/10.64898/2025.12.18.695326; this version posted December 22, 2025. The copyright holder for this preprint (which was not certified by peer review) is the author/funder, who has granted bioRxiv a license to display the preprint in perpetuity. It is made available under aCC-BY 4.0 International license.

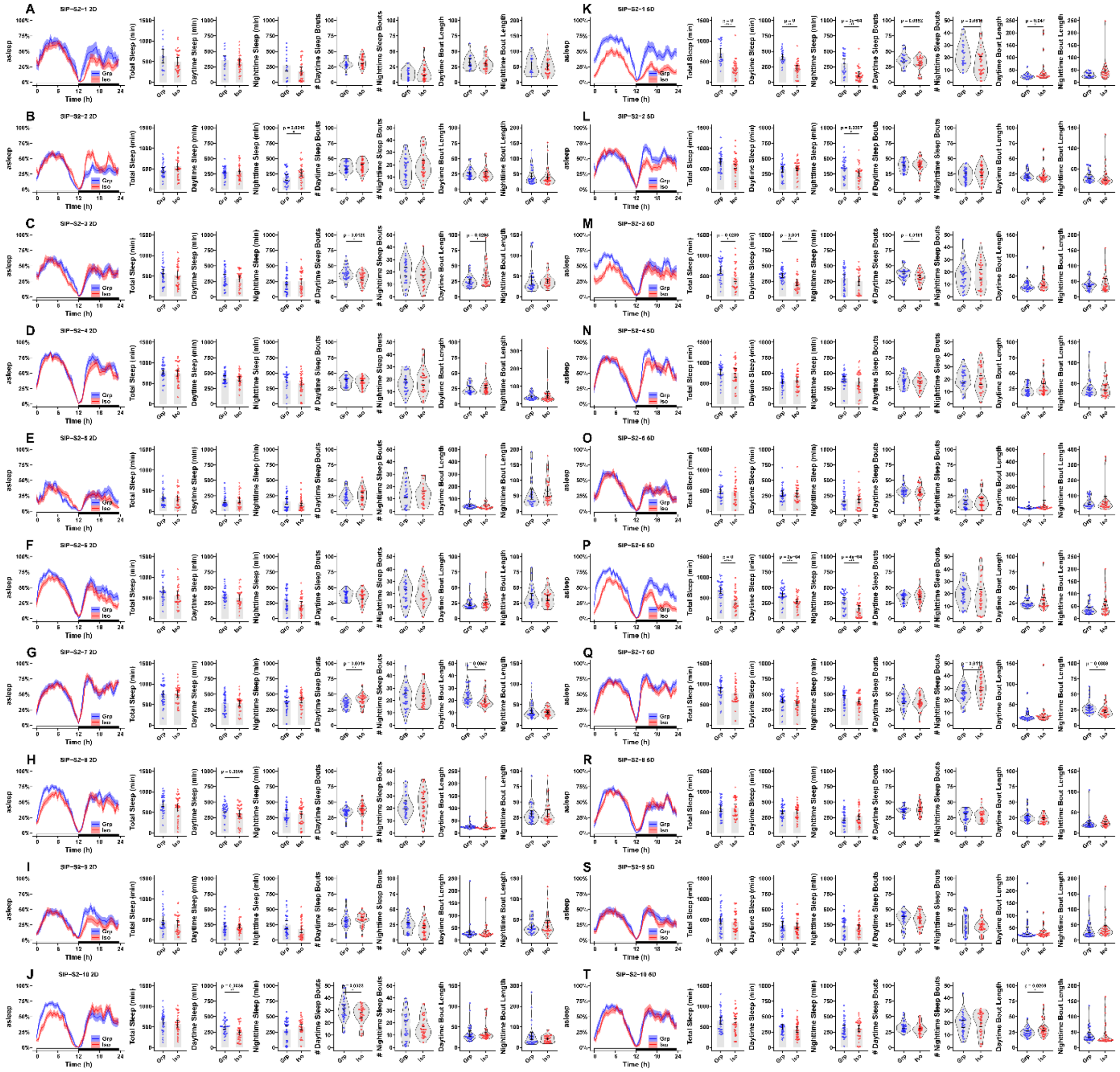

Supplementary Figure 6. Sleep profiles and parameters for SIP-S2 Strains. **(A–T)** SIP-S2 strains 1–10's sleep profiles, sleep parameters, and bout distributions after 2 days **(A–J)** or 5 days **(K–T)** of treatment. Statistical significance was assessed by two-sided unpaired *t*-tests (*$*P<0.05$, $**P<0.01$, $***P<0.001$, $****P<0.0001$*; n = 15–32).
